# Transcriptome Profiling of Hippocampus After Cerebral Hypoperfusion in Mice

**DOI:** 10.1007/s12031-023-02123-0

**Published:** 2023-06-02

**Authors:** Zengyu Zhang, Zimin Guo, Pengpeng Jin, Hualan Yang, Mengting Hu, Yuan Zhang, Zhilan Tu, Shuangxing Hou

**Affiliations:** 1 Department of Neurology, Shanghai Pudong Hospital, Fudan University Pudong Medical Center, Shanghai, 201399 China; 2 Shanghai Medical College, Fudan University, Shanghai, 200032 China; 3 Department of Chronic Disease Management, Shanghai Pudong Hospital, Fudan University Pudong Medical Center, Shanghai, 201399 China; 4 Department of Vascular Surgery, Shanghai Pudong Hospital, Fudan University Pudong Medical Center, 201399 Shanghai, China

**Keywords:** Chronic cerebral hypoperfusion, Bilateral carotid artery stenosis, Hippocampus, RNA-sequencing, Interferon signaling

## Abstract

Chronic cerebral hypoperfusion (CCH) is considered to be one of the major mechanism in the pathogenesis of vascular cognitive impairment (VCI). Increased inflammatory cells, particularly microglia, often parallel hypoperfusion-induced gray matter damage such as hippocampal lesions, but the exact mechanism remains largely unknown. To understand the pathological mechanisms, we analyzed hippocampus-specific transcriptome profiles after cerebral hypoperfusion. The mouse hypoperfusion model was induced by employing the 0.16/0.18 mm bilateral common carotid artery stenosis (BCAS) procedure. Cerebral blood flow (CBF) was assessed after 3-week hypoperfusion. Pathological changes were evaluated via hematoxylin staining and immunofluorescence staining. RNA-sequencing (RNA-seq) was performed using RNA samples of sham- or BCAS-operated mice, followed by quantitative real-time PCR (qRT-PCR) validation. We found that the 0.16/0.18 mm BCAS induced decreased CBF, hippocampal neuronal loss, and microglial activation. Furthermore, GSEA between sham and BCAS mice showed activation of interferon-beta signaling along with inflammatory immune responses. In addition, integrative analysis with published single-cell RNA-seq revealed that up-regulated differentially expressed genes (DEGs) were enriched in a distinct cell type of “microglia,” and down-regulated DEGs were enriched in “CA1 pyramidal,” not in “interneurons” or “S1 pyramidal.” This database of transcriptomic profiles of BCAS-hypoperfusion will be useful for future studies to explore potential targets for vascular cognitive dysfunction.

**Supplementary Information:**

The online version contains supplementary material available at 10.1007/s12031-023-02123-0.

## Introduction

Vascular cognitive impairment (VCI), due to hypoperfusion, accounts for at least 20% of cases of dementia caused by cerebrovascular diseases (Yin et al. 2022). The cause of VCI is multifactorial, including cerebral infarction, subcortical ischemia, multiple cerebral infarction, marginal zone ischemia, cerebral hemorrhage, and cerebral hypoperfusion (Saggu et al. 2016; O'Brien and Thomas 2015; van der Flier et al. 2018). Although the high disability rate of VCI imposes tremendous burden and economic cost to families and society (Kalaria, 2018), further researches such as disease-modifying treatments, reliable biomarkers for diagnosing diseases, and pathobiology of VCI are urgently needed (Iadecola et al. 2019). Accumulated evidence indicates that cerebrovascular pathology is the most consequential factor for dementia and that it interacts with neurodegenerative pathology in a complementary or synergistic way (Iadecola, 2013). Related studies have suggested that in most patients with VCI, imaging manifestations involve white matter hyperintensities, brain atrophy, lacunar infarction related to cerebrovascular abnormalities, cerebral microbleeds, subcortical and cortical infarction, etc. (Smith, 2017).

Chronic cerebral hypoperfusion (CCH), caused by moderate and persistent decreases in cerebral blood flow (CBF), is proved to be one of the most critical potential pathophysiological mechanisms in the development of vascular cognitive impairment (Wang, 2014, Washida et al. 2019). Relevant pathological studies have shown that in addition to the damage to the blood–brain barrier and white matter hyperintensities, the extent of hypoperfusion also greatly affects the degree of gray matter damage (Miki et al. 2009). Severe hypoperfusion can cause hippocampal lesions, including pyramidal neuron necrosis, apoptosis, and microglia activation (Washida et al. 2019). In the presence of long-term chronic ischemia, brain in situ immune glial cells will be activated to produce large amounts of informative substances and enzymes, which is an important pathophysiological aspect.

Nevertheless, the potential molecular mechanisms of hippocampal damage and glial cell activation in CCH remain poorly understood (Du et al. 2017). Numerous rodent models have been developed to mimic features of CCH; both the bilateral common carotid artery occlusion (BCAO) model and the bilateral common carotid artery stenosis (BCAS) model can simulate clinical patients with ischemic injury (Wan et al. 2017; Guo et al. 2021). Because of the inherent limitation of BCAO that CBF drops acutely after ligation of the arteries and resolves chronically, BCAS mouse model has been evaluated as one of the most germane rodent models of CCH (Tuo et al. 2021). Also, relevant literature reports indicate the BCAS model with no less than 0.18 mm coils is not enough to induce gray matter damage in mice, and the asymmetric ischemia in the left and right cerebral hemispheres caused by the 0.16/0.18 mm BCAS model is closer to the actual disease state in clinic. RNA-sequencing (RNA-seq), a powerful analytical tool in transcriptomics, is routinely used in genome-wide transcript analysis. Thus, it can be used to identify genes and pathways associated with hippocampal lesions after cerebral hypoperfusion.

Here, the 0.16/0.18 mm BCAS mouse model with significant hippocampal lesions and glial activation was established. Then, we performed hippocampus-specific bulk RNA-seq analysis, qRT-PCR validation, and integrative analysis with published single-cell RNA-seq, to investigate the molecular changes related to hippocampal damage caused by BCAS-hypoperfusion and further analyze the molecular mechanism, so as to provide potential targets for future research.

## Methods

### Animals

The animal experiments in this study were in compliance with the ARRIVE guidelines (Kilkenny et al. 2011). Adult male C57BL/6 J mice (9–10 weeks, weight 22–26 g) were provided by Beijing Vital River Laboratory Animal Technology. Mice were allowed to obtain food and water ad libitum and were housed in IVC cages under SPF conditions. After 1-week acclimation, the mice were randomly divided into 2 groups: the BCAS group and the sham group. A total of 49 mice were used in this study, 26 mice were in the BCAS group and 23 in the sham group, and 6 mice failed to survive the BCAS operation. The remaining 43 mice were used for the entire experiment.

### Bilateral Carotid Artery Stenosis Procedure

BCAS surgery was performed as described with minor modification (Miki et al. 2009; Shibata et al. 2004). Briefly, the mice were anesthetized with 2% isoflurane delivered with medical oxygen by a face mask. Then, both common carotid arteries (CCAs) were exposed and isolated from the vagus nerves through a midline incision. Microcoils, inner diameter of 0.16 and 0.18 mm (Anruike Biotechnology, Xi’an, China), were applied to the surgical procedure. As previously described, the 0.16 mm microcoil was wrapped around the right CCA (Zhou et al. 2022). One hour after the above operation was successful, the 0.18 mm microcoil was twined around the left CCA. Sham-treated animals were exposed to identical procedures with the exception that microcoils were not placed around the arteries. No intra-operative or post-surgical complications were observed in both groups of surviving mice. Mice were placed back to the normal cage to recover with unrestricted access to food and water.

### Laser Speckle Contrast Imaging

To determine the impact of hypoperfusion on cerebral blood flow (CBF), laser speckle contrast imaging (LSCI) was used 3 weeks following surgery (RFLSI III, RWD, China) as described previously (Mao et al. 2017). Briefly, each mouse was anesthetized with isoflurane, and the head was placed in a stereotaxic frame in a prone position. An incision was made along the midline of the scalp, and the skull was exposed. Then, a whole-brain scan was performed using the LSCI. Regions of interest (ROIs) were manually selected, and the data were analyzed using the LSCI_V 1.0.0 software (RWD, China) to assess CBF changes. Following the measurement, the skin incision was gently sutured. Throughout the experiment, body temperature was maintained at 36.5–37.5℃.

### Tissue Preparation

At three weeks post surgery, mice were anesthetized with 2% isoflurane and decapitated. Then, take out the brain, fix it in 4% formaldehyde in PBS at 4℃ overnight, and dehydrate to sink in 30% glucose at 4℃. Coronal slices at 35 µm thickness were prepared using a freezing microtome (CM 1900, Leica, Germany).

### Hematoxylin Staining

Five consecutive representative sections in each brain were selected for hematoxylin staining. We performed hematoxylin staining using the Solarbio kit (G1120) with minor modification because of these changes of phenotypes which could be captured without cytoplasm staining by eosin (Cao et al. 2018; Liu et al. 2018). For the staining process, brain sections were fixed in methanol and stained with hematoxylin. Rinse the sections quickly with 1% HCl ethanol solution to remove excessive background staining, following the treatment with differentiation buffer. The slides were passed through 70% alcohol and anhydrous alcohol and mounted with xylene mountant. Images were captured by the Olympus slide scanner (VS120-L100). Five brain slices from 5 mice in each group underwent a whole-brain scan. Field of view of the left or right hemisphere was selected. In addition to apoptosis and necrosis in related brain regions, the infarct focus is characterized by the accumulation of mononuclear cells or microglia nuclei. The area of infarction is measured by the OlyVIA (Olympus) software. For the assessment of infarction, area was manually outlined (“measures a freehand polygon”), and the measure was noted for each region. Then, the software automatically calculated the aimed area values within the selection. The formula for calculating the section of cerebral infarction area (%) was (infarct area of the ipsilateral hemisphere)/(whole-brain area) × 100%.

### Immunofluorescence Staining

Tissue preparation was performed as described above. For immunofluorescence staining, selected sections in the state of free-floating were washed in 1 × PBS and incubated with a blocking buffer (1% Triton X-100 in PBS containing 5% normal goat serum (Thermo Fisher, 16,210,064) for 1 h. Sections were treated with primary antibody against Iba-1 (Abcam, ab178846) in blocking solution at 4 °C. These brain sections after the overnight incubation were then incubated with secondary antibody (Goat anti-rabbit Alexa Fluor 594, Jackson, 111–585-003) for 1 h, followed by DAPI (Thermo Fisher, 62,248, 1:10,000 dilution) counterstaining for 15 min. Sections were obtained by an Olympus slide scanner (VS120-L100). High resolution images were captured from the confocal microscopy (SP8 LSCM, Leica, Germany). Images were processed using the ImageJ software. A whole-brain scan was seen in each of the five brain slices that were selected for each animal. Field of view of the left or right hemisphere was selected. Compared with static microglia, activated microglia are characterized by stronger fluorescence intensity and higher local distribution density (Lim et al. 2018). And “Freehand polygon” of “Tool windows” in the OlyVIA software was used to manually define the area with activation of microglia and the whole section area. And the software automatically calculated the aimed area values within the selection. The area values were automatically calculated by the software. The relative activation area (%) was defined as (activation area of the hemisphere)/(whole-brain area) × 100%.

### RNA Extraction

Total RNAs were extracted from the entire right hippocampus by using TRIzol Reagent (Invitrogen, USA). RNA concentration and purity were measured by NanoDrop 2000 (Thermo Fisher Scientific). The RNA sample was stored at − 80℃ before use.

### RNA-seq and Data Analysis

Three RNA samples from each group were randomly selected for RNA-seq experiments. The cDNA library was constructed using a NEBNext Ultra™ RNA Library Prep Kit for Illumina (NEB, USA) following manufacturer’s instructions and was successfully sequenced on an Illumina HiSeq™ sequencing platform with a pattern of PE150. The raw data was obtained in FASTQ format, and FastQC was used for quality control (the quality control of RNA-seq data is presented in Fig. S1 and Table S2). Data preprocessing was carried out using Trim-galore to obtain high-quality clean reads. In this step, adapter sequences, low quality reads, and too short reads adapter were filtered. All the downstream analyses were based on the clean data with high quality. Next, sequencing reads were aligned to annotated RefSeq genes in the mouse reference genome (UCSC mm10) using HISAT2. SAMtools (v1.9) was then used to convert, sort, and index alignments. FeatureCounts v1.6.3 was used to count the reads numbers mapped to each gene, and then, FPKM of each gene was calculated based on the length of the gene and reads count mapped to this gene. GSEA software (version 3.0) was used for the gene set enrichment analysis. The DESeq2 was used for differential expression analysis of the BCAS group and sham group. The level with an adjusted *P* value < 0.05 found by DESeq2 and |Log2fold change|> 1 was set to filter differential expression genes (DEGs). Gene Ontology (GO) analysis is a functional analysis associating DEGs with GO categories. Then, GO enrichment analysis of DEGs was performed by the ShinyGO 0.76.3, and GO terms with adjusted *P* value less than 0.05 were considered significantly enriched by DEGs. The sequence data (FASTQ files) were deposited under the accession code GSE223580.

### Quantitative RT-PCR

RNA was extracted from the whole right hippocampus tissue using the TRIzol Reagent as described previously. Reverse transcription was performed using Evo M-MLV RT Kit (AG11711, Accurate Biology), and SYBR® Green Premix qPCR Kit (Thermo Fisher, Applied Biosystems QuantStudio 5) was used to perform qRT-PCR (AG11718, Accurate Biology). The primer sequences are listed in Table S2. All relative gene expression analyses were performed using the 2^−ΔΔCt^ and were normalized to GAPDH as the reference gene.

### Integrative Analysis of Bulk RNA-seq and Published scRNA-seq

Analysis was carried out as we described previously (Zhang et al. 2022). Dimensionality reduction was performed using the UMAP algorithm. Firstly, cell clusters were generated with Seurat in the UMAP plot using the published scRNA-seq data (GSE60361) (Zeisel et al. 2015). Then, we performed cell-type enrichment analysis by using DEGs of our hippocampus-specific bulk RNA-seq and the published scRNA-seq data. The full list of DEGs was divided into two lists: a list of “BCAS-induced up-regulated genes” (up_genes) and a list of “BCAS-induced down-regulated genes” (down_genes). Moreover, Seurat’s DoHeatmap visualizes the expression value of scRNA-seq for the individual gene in the two lists. Of note, only those genes detected in scRNA-seq were shown. Furthermore, the Seurat function FindAllMarkers was employed to identify marker genes of each cell cluster in scRNA-seq data with the parameters by default. And enrichment scores of genes in these two lists in each cell cluster were generated using R package GeneOverlap. Lastly, cell-type deconvolution analysis was performed using the *AUCell* algorithm from the open-source R package at https://github.com/chuiqin/irGSEA.

### Statistical Analysis

All experiments and data analysis were conducted under investigator-blinded conditions. Statistical analyses were performed using Prism 9 for Windows (GraphPad Software). Paired *t* test was used for the data comparisons of 0.18 mm side and 0.16 mm side in BCAS group samples (two-tailed) while an unpaired *t* test was performed (two-tailed) in the comparisons made between the sham and BCAS groups. All data were expressed as mean ± SEM, and significance level was set as *P* < 0.05. ^*^P < 0.05, ^**^P < 0.01, and ^***^P < 0.001.

## Results

### Cerebral Blood Flow and Histological Changes After Cerebral Hypoperfusion

**Fig. 1:**
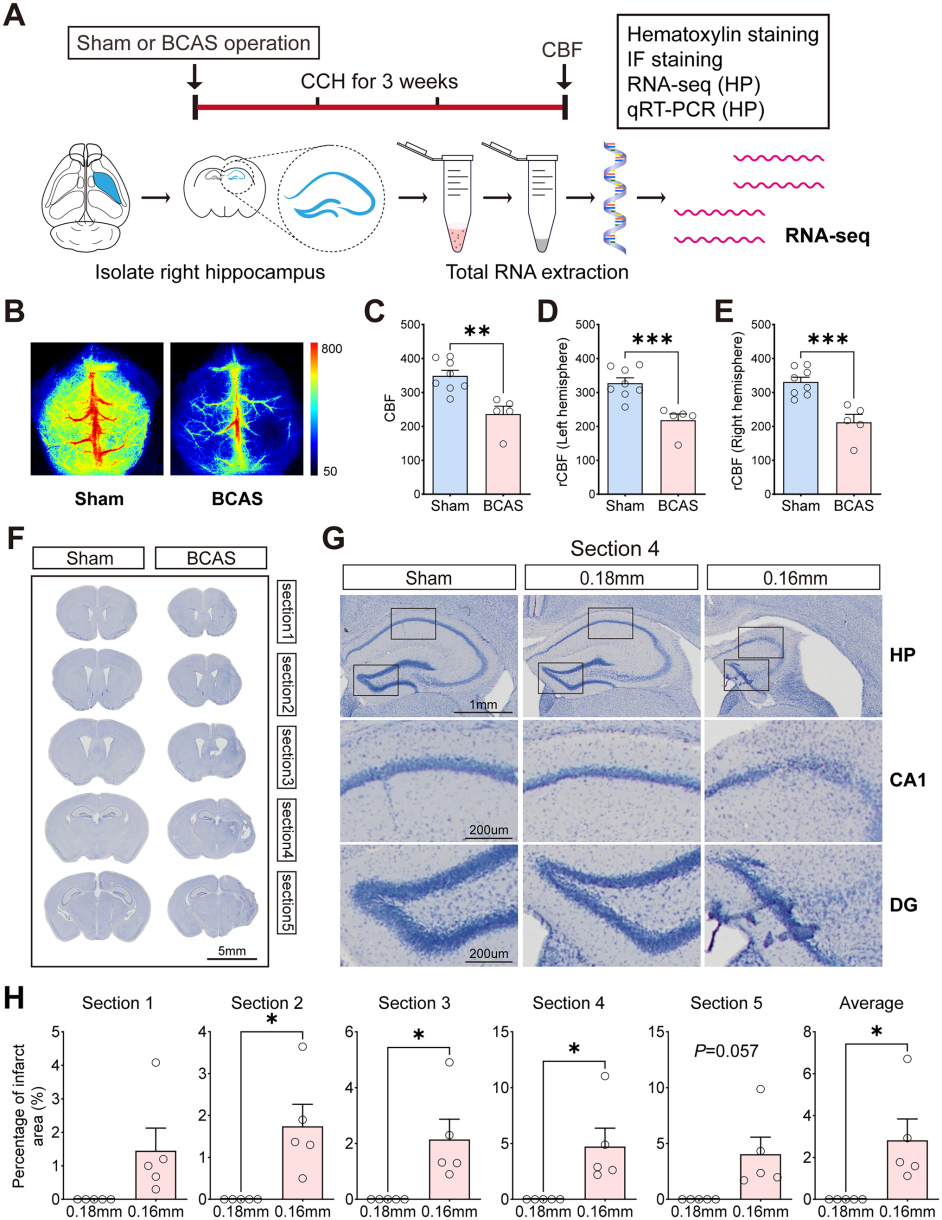
Cerebral blood flow (CBF) and histopathological alterations after 3-week cerebral hypoperfusion. **A** The experimental flowchart. BCAS, bilateral carotid artery stenosis; CCH, chronic cerebral hypoperfusion; IF, immunofluorescence staining; HP, hippocampus. **B** Representative images of laser speckle CBF at 3 weeks following BCAS or sham operation. Bar plots for whole brain (**C**), left hemisphere (**D**), and right hemisphere (**E**) CBF in sham and BCAS mice (*n* = 5–8 mice per group). **F** Hematoxylin-stained coronal brain sections show brain injury after BCAS-hypoperfusion. **G** Representative images of hematoxylin staining in the hippocampal regions. CA1, hippocampal CA1 area; DG, dentate gyrus. **H** Bar plots showing the percentage of infarct area in each section on the 0.16 mm and 0.18 mm sides of the BCAS group. Average, each point represents the mean value of five sections of individual mice. Data are expressed as mean ± SEM. Paired *t* test (two-tailed), ^*^P < 0.05 versus 0.18 mm side, *n* = 5 mice for per group

In this study, there was a significant reduction of CBF on the left and right sides of the brain 3 weeks after BCAS-hypoperfusion, which was consistent with prior studies (Zhou et al. 2022) (Fig. 1B–E). The result demonstrated that BCAS mice using 0.16/0.18 mm microcoils led to an approximate 36% decrease in the average CBF compared to the right hemisphere of sham mice. To evaluate histopathological alterations of the brain after cerebral hypoperfusion, we performed hematoxylin staining of five different sections (Fig. 1F). Hippocampal atrophy on the right hemisphere was present. Furthermore, profound neuronal loss was evident in the CA1 and DG regions of the hippocampus (Fig. 1G). Then, we compared the percentage infarct sizes of the 0.18 mm and 0.16 mm stenosis sides of five sections in BCAS mice. The mean percent infarct size of each mouse was also compared. Results showed that BCAS mice exhibited significantly increased infarct areas on the 0.16 mm side when compared to the 0.18 mm side (Fig. 1H).

### Hippocampal Lesions with Microglial Activation Following Cerebral Hypoperfusion

**Fig. 2:**
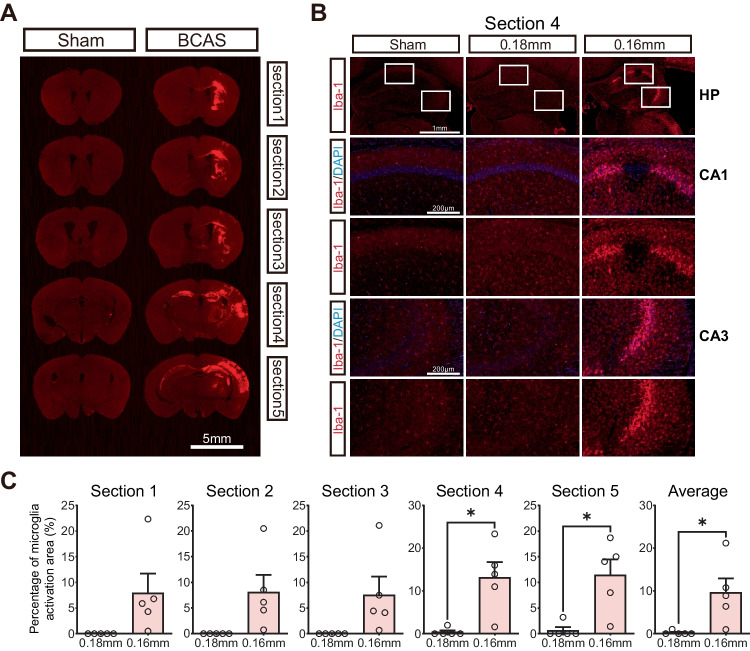
Microglial activation induced by BCAS-hypoperfusion 3 weeks post operation. **A** Representative Iba-1 stained coronal sections of the brains from sham and BCAS mice. **B** Representative images of Iba-1 labeled sections showing obvious microglial activation in hippocampal CA1 and CA3 regions. **C** Bar plots of percentage of microglial activation area of each section in BCAS mice. Average, each point represents the mean value of five sections of individual mice. Data are expressed as mean ± SEM. Paired *t* test (two-tailed), ^*^P < 0.05 versus 0.18 mm side, *n* = 5 mice for per group

Immunofluorescence staining with the microglial marker Iba-1 was used to identify the injury areas with reactive microglial/macrophages. We first compared Iba1-stained coronal sections of the brains from sham and BCAS mice (Fig. 2A). We found that profound microglial activation was seen within the infarct lesions, including hippocampal CA1 and CA3 regions of the 0.16 mm side in BCAS mice (Fig. 2B). In addition, there was significantly increased microglial activation on the 0.16 mm side compared with the 0.18 mm side of the BCAS group by comparing the percentage of microglial activation of five sections as well as the mean percent activation area of individual mice (Fig. 2C). And these observations were consistent with the previous literature (Qin et al. 2017). Therefore, 0.16/0.18 mm BCAS could induce significant histological injury and microglial activation of the brain, involvement of the hippocampus region. Our results are consistent with prior observations and provide a comprehensive basis for studying neuropathological changes in whole hippocampus and in all hippocampal subfields following cerebral hypoperfusion.

### Transcriptional Activation of IFN-Regulated Genes in Hippocampus After Cerebral Hypoperfusion

**Fig. 3:**
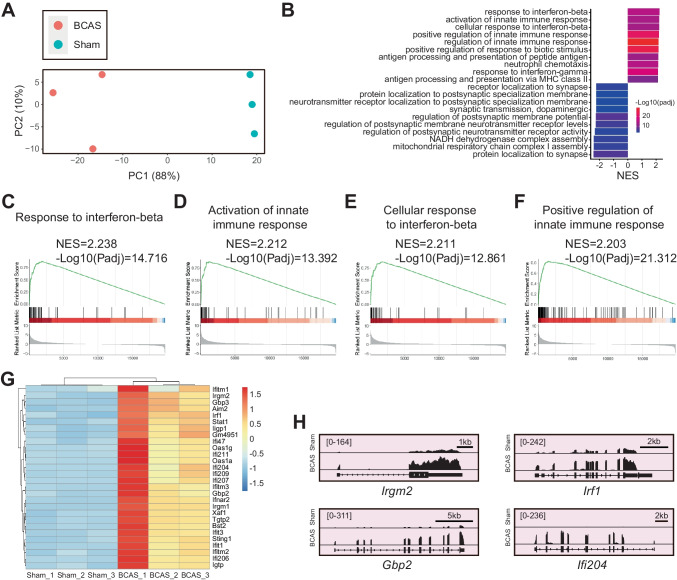
Hippocampus-specific gene set enrichment analysis (GSEA) for transcriptome following cerebral hypoperfusion. **A** Principal component analysis (PCA) plot of RNA-seq datasets obtained from sham and BCAS mice. *N* = 3 for per group. Note the whole right hippocampus used here. **B** Up-regulated and down-regulated functional pathways analyzed by GSEA (BCAS vs. sham). NES, normalized enrichment score. Notice high enrichment for interferon-beta (IFN-β) signaling pathways. **C**–**F** Enrichment plots showing positive enrichment for IFN-β-related immune pathways. **G** Heatmap of up-modulated genes involved in the IFN-β signaling. **H** IGV map tracks for representative genes in the up-regulated pathways. Notice increases of RNA-seq signal for IFN-regulated genes (*Ifi204*, *Gbp2*, *Irgm2*, *Irf1*) in the BCAS group compared with the sham group

We focused our attention on hippocampus-specific transcriptional changes induced by 0.16/0.18 mm BCAS. The RNA-seq experiments were performed using three biological replicates at 3 weeks post procedure. Principal component analysis (PCA) confirmed clear separation between the BCAS and sham groups (Fig. 3A). PCA scores were 88% for PC1 and 10% for PC2, indicating the unique transcriptome signature of the hippocampus after BCAS-hypoperfusion. Gene set enrichment analysis (GSEA) was used to analyze the signaling pathway enrichment in these groups. Results showed multiple extremely up-regulated pathways important in interferon (IFN)-mediated biological processes in BCAS mice compared with sham mice. The top four were “response to interferon-beta (Padj: 1.92E-15; NES: 2.24),” “activation of innate immune response (Padj: 4.06E-14; NES: 2.21),” “cellular response to interferon-beta (Padj: 1.38E-13; NES: 2.21),” and “positive regulation of innate immune response (Padj: 4.88E-22; NES: 2.20)” (Fig. 3B–F, Table S3). In addition, consistent with pathological neuronal loss in the hippocampal region, GSEA also showed several significantly important down-regulated pathways related to neuronal activity in BCAS mice. The top four were “protein localization to synapse (Padj: 3.15E-07; NES: − 2.42),” “mitochondrial respiratory chain complex I assembly (Padj: 3.42E-05; NES: − 2.40),” “NADH dehydrogenase complex assembly (Padj: 3.42E-05; NES: − 2.40),” and “regulation of postsynaptic neurotransmitter receptor activity (Padj: 2.15E-04; NES: − 2.33)” (Figs. 3B, S2 and Table S3). Further analysis of these pathways revealed that the transcriptional differences were evident especially for IFN-regulated genes (IRGs), as depicted in the heatmap and IGV map of the interferon-beta pathway (Fig. 3G, H). Together, our data suggested that transcriptional activation of IRGs and transcriptional repression of neuronal activity genes occur after chronic hypoperfusion.

### Quantitative RT-PCR Validation of Transcriptional Changes Detected by RNA-seq

**Fig. 4:**
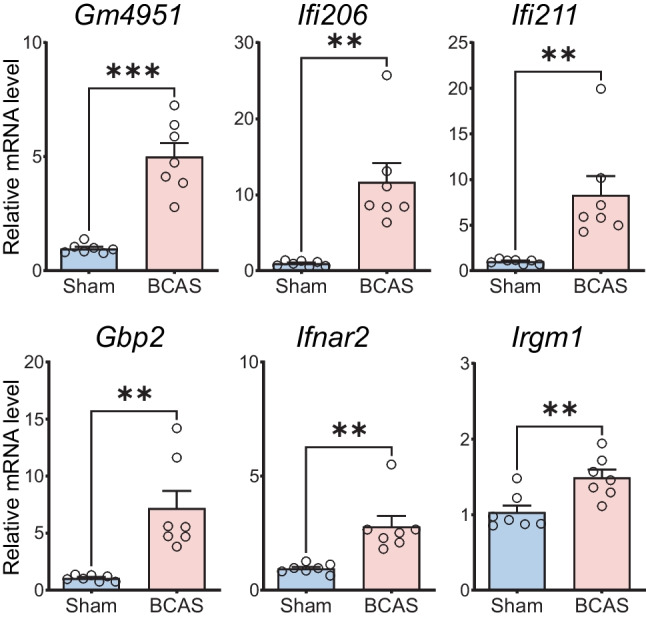
Validation of gene expression changes by quantitative RT-PCR. The relative mRNA expression levels of representative genes in the whole right hippocampus of sham and BCAS mice (gene expression was normalized by the housekeeping gene *Gapdh*). Up-regulated genes involved in “response to interferon-beta” are shown. Data are expressed as mean ± SEM. Unpaired *t* test (two-tailed), ^**^P ˂ 0.01 and ^***^P ˂ 0.001 versus sham, *n* = 7 mice for each group

We performed qRT-PCR to detect the expression of genes highly enriched in the IFN-related immune response pathways, including “response to interferon-beta” (*Gm4951*, *Ifi206*, *Ifi211*, *Gbp2*, *Ifnar2*, *Irgm1*). Compared with the sham mice, the BCAS mice exhibited increased mRNA levels of these genes in the hippocampus. These findings were consistent with the results from the RNA-seq transcriptome analysis (Fig. 4).

### Gene Ontology Enrichment Analysis of Differentially Expressed Genes Following Cerebral Hypoperfusion

**Fig. 5:**
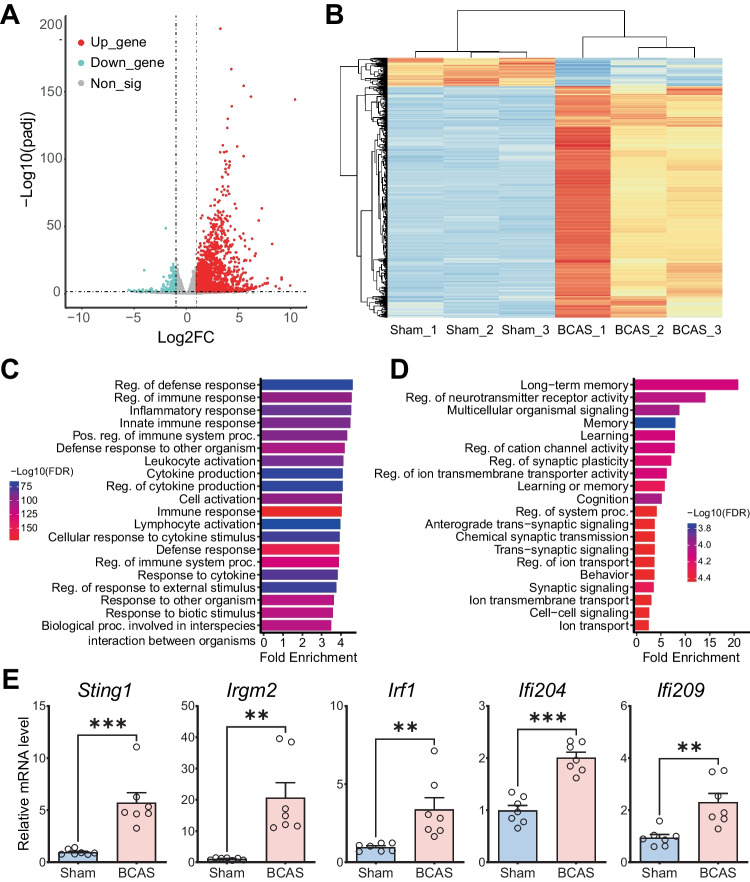
Gene expression changes and Gene Ontology (GO) enrichment analysis of differential expression genes (DEGs) after 3-week cerebral hypoperfusion. **A** Volcano plots of DEGs in the hippocampus (BCAS vs. sham). Note the whole right hippocampus used here. DEGs significantly up-regulated and down-regulated by RNA-seq analysis (log2FC cutoff of 1, adjusted *P* value cutoff of 0.05). Each dot corresponds to an expressed gene; DEGs are highlighted in red (up-regulated) and blue (down-regulated) colors. **B** Sample correlation heatmap. **C** Bar plot showing enriched GO biological process terms of up-regulated genes. **D** Bar plot showing enriched GO biological process terms of down-regulated genes. **E** Real-time RT-PCR validation for the up-regulated genes in “regulation of defense response.” *N* = 7 for each group. Data are expressed as mean ± SEM. Unpaired *t* test, two-tailed, ^**^P < 0.01 and.^***^P < 0.001

Here, gene sets with substantial alterations were analyzed. Standard thresholds (a |log2FC|> 1 combined with an adjusted *P* value (FDR) < 0.05) were used to define sets of DEGs. In these two groups, a total of 1854 DEGs were identified in the hippocampus, including 1641 (88.5%) significantly up-regulated DEGs and 213 (11.5%) significantly down-regulated DEGs (Fig. 5A, Table S4). Clustering, drawn above the heatmap, demonstrated that the samples of BCAS group were very similar to each other but showed complete separation from the sham group samples (Fig. 5B). And the majority of the DEGs were up-regulated following BCAS-hypoperfusion. In addition, GO enrichment analysis was performed according to the DEGs. The top 20 biological process (BP) GO terms for up-regulated and down-regulated DEGs were identified (Fig. 5C, D). The results demonstrated that immune-related biological processes including “regulation of defense response,” “regulation of immune response,” “inflammatory response,” and “innate immune response” were significantly enriched and up-regulated (Fig. 5C). Also, we found extremely important down-regulated signal pathways associated with neurons after BCAS-hypoperfusion. Of these, the most significantly associated pathways were “long-term memory,” “regulation of neuro-transmitter receptor activity,” and “multicellular organismal signaling” (Fig. 5D). We further performed real-time RT-PCR on isolated hippocampus and validated the increase of some up-regulated IRGs in BCAS mice compared to sham mice (Fig. 5E). Collectively, GO enrichment analysis of the DEGs in the hippocampus indicated that most of the significantly enriched GO terms after chronic hypoperfusion injury were positive regulation of inflammatory and immune responses.

### Hippocampus-Specific DEGs Induced by BCAS-Hypoperfusion Were Enriched in a Distinct Cell Type of the Brain

**Fig. 6:**
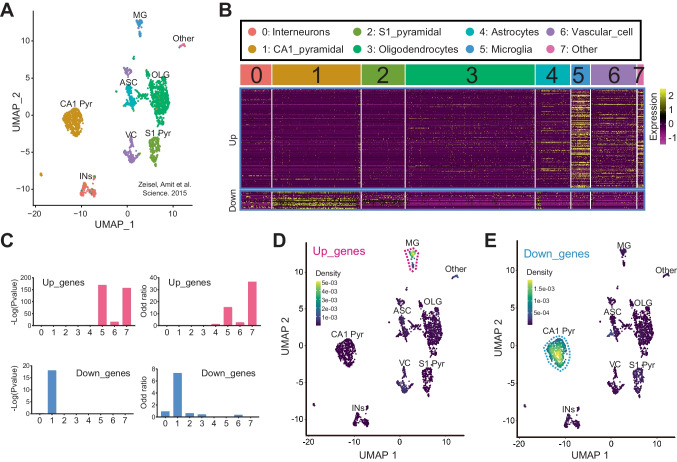
Enrichment of cerebral hypoperfusion-induced DEGs in a distinct cell type of the brain. **A** The UMAP plot visualizing clustering of single cells colored by cell types using published single-cell RNA-seq data (Zeisel et al. 2015, Science). **B** Seurat’s DoHeatmap shows the individual gene expression level of DEGs in each cell of the published single-cell RNA-seq (GSE60361). The eight cell clusters (cluster 0 to cluster 7) represent eight classes of brain cells. **C** The bar plots show the gene set enrichment score and odds ratio (95% CI) of “up_genes” and “down_genes” in each cell type of the brain. **D**, **E** Density scatterplots visualize cell-type enrichment for **D** “up_genes” and **E** “down_genes” using *AUCell* R package (https://github.com/chuiqin/irGSEA). Visualized genes are DEGs from the hippocampus bulk RNA-seq in the current study. Notice the enrichment of up-regulated genes in cluster 5 of “MG” and down-regulated genes in cluster 1 of “CA1 Pyr.” Ins, interneurons; CA1 Pyr, CA1 pyramidal; S1 Pyr, S1 pyramidal; OLG, oligodendrocytes; ASC, astrocytes; MG, microglia; VC, vascular cells; Other, other cell types

Previous studies revealed different cell types in the mouse brain using single-cell RNA-seq under normal or pathological conditions (Zeisel et al. 2015; Zheng et al. 2022; Li et al. 2022). As shown in Figs. 6A and S3, we identified eight transcriptionally distinct clusters via detection of known cell type markers using a published scRNA-seq dataset (GSE60361) of mouse cortical and hippocampal cells (Zeisel et al. 2015). The eight major classes of cells included “interneurons” (cluster 0), “CA1 pyramidal” (cluster 1), “S1 pyramidal” (cluster 2), “oligodendrocytes” (cluster 3), “astrocytes” (cluster 4), “microglia” (cluster 5), “vascular cells” (cluster 6), and “other” (cluster 7). It should be added that vascular cells are composed of three cell types: vascular endothelial cells, vascular smooth muscle cells, and perivascular cells. Then, we visualized the expression of our hippocampus-specific DEGs in single cell of this published scRNA-seq (GSE60361). And we found a distinct distribution pattern in “microglia” for the “BCAS-induced up-regulated genes” (up_genes) and “CA1 pyramidal” for the “BCAS-induced down-regulated genes” (down_genes) (Fig. 6B). Furthermore, gene set enrichment analysis also showed that the set of up-regulated genes was significantly enriched in “microglia” (*P* = 5.61e-170, odds ratio 15.4). In contrast, the down-regulated gene set was enriched in “CA1 pyramidal” (*P* = 8.17e-19, odds ratio 7.3), not in “interneurons” or “S1 pyramidal” (Fig. 6C), with our analysis results matching the pathological observations. In addition, we did cell-type enrichment analysis using the *AUCell* algorithm in irGSEA. As shown in Fig. 6D, E, a distinct “BCAS-induced up-regulated genes” distribution pattern in “microglia” was observed. And we also noticed a unique enrichment for the “BCAS-induced down-regulated genes” in another “CA1 pyramidal” class. Taken together, our results indicated that distinct cell type in the hippocampus responded to BCAS-hypoperfusion differentially with the transcriptional activation occurring primarily in “microglia” as well as repression in “CA1 pyramidal.”

#### The Elevated IFN-β Signaling Molecule Expression Caused by BCAS-Hypoperfusion Is Correlated with Microglial Activation

**Fig. 7:**
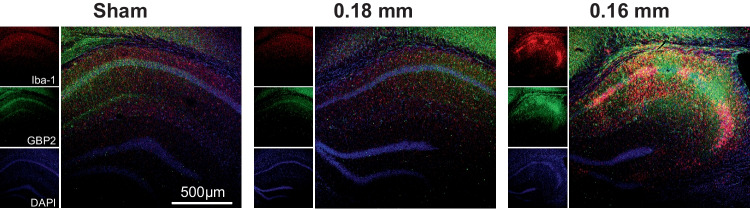
The altered microglia phenotype was accompanied by elevated expression of IFN-β signaling marker GBP2. The representative confocal image of GBP2/lba-1 co-staining of hippocampal region in both sham and BCAS groups (× 10)

From the above results, we can conclude that the change of gene expression induced by BCAS-hypoperfusion is closely related to the activation of microglia and that BCAS-induced up-regulated genes are significantly enriched in IFN-β-mediated signals. As the critical protein induced by IFNs, guanylate-binding protein 2 (GBP2) plays a significant role in the IFN-β signaling response. Therefore, we conducted double immunofluorescence labeling for GBP2 and Iba-1 to investigate the expression and cellular localization of GBP2 after BCAS-hypoperfusion. Representative confocal images of the hippocampal region were captured, and colocalization of GBP2 with partially activated microglia was observed around the lesion site on the 0.16 mm stenosis side of the BCAS group (Fig. 7). Interestingly, microglial activation was accompanied by increased production of GBP2, and GBP2 was activated in microglia due to BCAS-hypoperfusion.

## Discussion

The present study examined the effect of chronic cerebral hypoperfusion on hippocampal lesions and glial activation. Then, we performed RNA-seq analyses to investigate hippocampus-specific transcriptomic changes after 3 weeks of BCAS-hypoperfusion. Our initial findings suggested that large-scale transcriptional activation in hippocampus was observed in response to chronic ischemic injury, in particular, IFN-regulated genes. We also found that up-regulated genes were closely related to interferon-beta and inflammatory immune pathways, and down-regulated genes were associated with neuronal activity pathways. Remarkably, integrative analysis with a published scRNA-seq dataset demonstrated that up-regulated DEGs were significantly enriched in a distinct cell type of “microglia.” Also, the down-regulated DEGs were highly enriched in the “CA1 pyramidal.” Our results have the potential to set the stage for the research identifying and developing effective targets for VCI and other ischemic injury related diseases.

Gray matter dysfunction is reported to be a major neuropathological feature of many central nervous system diseases (Nishio et al. 2010). Nevertheless, the majority of basic researches on chronic cerebral hypoperfusion have focused on the pathological mechanisms of white matter (Cao et al. 2022; Suzuki et al. 2021; Shibata et al. 2007). Indeed, in addition to white matter lesions, chronic hypoperfusion is also related to gray matter damage such as cortical and hippocampal lesions in the severe cerebral hypoperfusion state. The hippocampal region of the mammalian brain is essential for higher brain functions, such as learning and memory, which are closely associated with activity-dependent synaptic plasticity (Mardones et al. 2016). Multiple rodent models characterizing features of cerebral hypoperfusion with hippocampal lesions have constructed (Nishio et al. 2010, Wang, 2014, Somredngan and Thong-Asa 2018). Notable in this respect was the establishment of the 0.16/0.18 mm BCAS, with this chronic hypoperfusion model more relevant to the pathophysiology of VCI and more closely resembling the in vivo state (Miki et al. 2009). This mouse model is characterized by several VCI-related neuropathological features including reduced CBF, cognitive impairment, gray matter lesions involving hippocampal region, and chronic neuroinflammation (Miki et al. 2009). Another most recent study also showed that BCAS using 0.16/0.18 mm microcoils is an excellent model for studying the mechanism and developing therapy of vascular cognitive dysfunction (Zhou et al. 2022). Significantly, our previous study showed that the 0.16/0.18 mm BCAS model can lead to cerebral gray matter damage, including hippocampal lesions. Microglia were markedly activated with phagocytosis-like phenotype changes. Activated microglia can cause secondary neuronal injury, and our study established the basis for subsequent neuropathological studies of gray matter injury caused by CCH (Zhang et al. 2023). Furthermore, in order to evaluate functional changes after prolonged cerebral hypoperfusion in the 0.16/0.18 mm BCAS model, we utilized a Morris water maze to test spatial cognitive function between the sham and BCAS groups. The results indicated that mice in the BCAS group showed impaired spatial learning ability compared with the mice in the sham group postoperatively. Therefore, we chose this BCAS model for transcriptome profiling in order to capture the key genes and pathways associated with CCH.

One of the major strengths of this study was the investigation of hippocampus-specific gene expression changes following BCAS-hypoperfusion by RNA-seq analysis. Our GSEA analysis revealed that in the hippocampus of BCAS mice, the up-regulated genes were extremely enriched in the interferon-beta signaling (Fig. 3B–H, Table S3). Also, the confirmation by qRT-PCR showed that the expression levels of DEGs were consistent with the RNA-seq data, suggesting that the RNA-seq results were reliable. We concluded that IFN-mediated neuroimmune signaling exerts a crucial regulatory effect on hippocampus in response to chronic cerebral hypoperfusion. IFN-signaling pathways are critical intermediates of cellular signaling pathways and can be divided into three immune subtypes (Harmon et al. 2022). Of note, type I (IFN-α and IFN-β) exhibits proinflammatory activity, and type II (IFN-γ) has anti-inflammatory effect. Interestingly, in our findings, previously unreported IRGs were identified according to the hypoperfusion-induced hippocampus-specific transcriptomes. Although type I IFN signaling has been suggested to play an important role in fighting central nervous system infections, little is known about its underlying effect on chronic ischemic injury (Chen et al. 2022). In this study, we revealed that *Irf1* was significantly up-regulated in BCAS mice and contributed to IRGs in immune system regulation (Fig. 3G, H). The interferon regulatory factor-1 (IRF1), a transcription factor (TF), is known as an important regulator of genes involved in multiple pathophysiological processes, such as cellular response to programmed cell death and inflammation (Huang et al. 2020; Jefferies, 2019; Liu et al. 2020). And IRF1 was initially considered to be an important regulator of IFNs and IRGs. The main effect of IRFs on immune responses is mainly linked to the control of the type I interferon system, which must be not only rapidly activated to mount an immune response but also tightly regulated to avoid adverse effects (Mancino and Natoli 2016). Activation of IRF1 is controlled by phosphorylation events that result in the formation of homodimers that are transcriptionally active (Tamura et al. 2008; Negishi et al. 2018). Therefore, we speculated that IRF1-mediated immune responses contributed to the transcriptional activation of IRGs after BCAS-hypoperfusion in our study. In future studies, it would be of interest to investigate how these IFN-related immune responses participate in the mechanics of chronic cerebral hypoperfusion.

While no studies have carefully examined the interferon-beta responses in the hippocampal region of BCAS-hypoperfusion mice so far, activation of IFN-related signaling pathways has been confirmed in multiple rodent models of neurological disorders (Kong et al. 2022; Harmon et al. 2022). For example, the guanylate-binding protein 2 (GBP2), one member of GBP family, is induced by IFNs. Within the GBP family of proteins, many are highly up-regulated by IFN signaling. And the majority of GBP genes such as *Gbp2* have been used as markers for IFN responsiveness in both cells and organisms (Messmer-Blust et al. 2010). Recent studies demonstrated that colocalization was also observed for GBP2 (IFN-induced protein) and Iba1 (microglia marker) through double immunofluorescence analysis in the brain cortex in a rat model of traumatic brain injury, suggesting that GBP2 might play a vital role in microglia activation process following brain damage (Miao et al. 2017). It thus seems like up-regulation of IFN-signaling pathways would also be expected in the hippocampus after cerebral hypoperfusion (Fig. 3G, H). In the “Results” section, we present our observations by integrative analysis and found that hippocampus-specific DEGs induced by BCAS-hypoperfusion play an essential role in microglia activation (Fig. 6B–E). Interestingly, we also confirmed that up-regulation of GBP2 was closely associated with microglial activation in hippocampal region of mouse brain following BCAS-hypoperfusion (Fig. 7). These studies emphasize the importance of IFN-β signaling molecule in neuroinflammation, with GBP2 being identified as a new player involved in microglial phenotype modulation in response to a variety of inflammatory conditions. Therefore, a better understanding of the changes in type I IFN signals could help to clarify the cellular and molecular mechanics of CCH-induced brain injury.

Immune responses accompanying cerebral ischemic injury involve microglia, astrocytes, T cells, etc., among which the brain’s primary immune sentinels are microglia cells. The microglia act as the resident macrophages of the brain and play essential roles in the hub of intercellular communication in innate immunity and neuroinflammatory pathologies (Mao et al. 2017). Microglial cells are rapidly activated in response to infection, inflammation, and injury, associated with the expression of pro/anti-inflammatory genes and secretion of cytokines (Wang et al. 2018). Both in vitro and in vivo experiments showed that loss of BIN1 (the key regulators of inflammation) impaired the ability of microglia to mount type I interferon responses to proinflammatory challenge, particularly the up-regulation of critical IFN-signaling pathway genes (Sudwarts et al. 2022). Moreover, microglia are considered to be critical in response to the IFN-signaling response, acting via the induction of cytokines (primarily type I IFNs), and have been reported in multiple studies to play a role in modulating neuroinflammation. And the researchers also found that microglia-specific *Ifnar1* deletion prevented post-synaptic terminals loss by selective engulfment. Furthermore, curing brain disorders through precision medicine is the overarching goal of a new wave of molecular and genomic therapies (Liu et al. 2019; Sudwarts et al. 2022). Encouragingly, a variety of approaches for genetic targeting of microglia in mice have been tested, including the targeting for CD11b and CD11c, the enzyme lysozyme M (LysM), F4/80, colony-stimulating factor 1 receptor (CSF1R), Iba1, and CX_3_CR1 (Wieghofer et al. 2015). In addition, researches have shown that delivery of drugs using novel nanoparticle technologies offers a potential strategy for targeting glia in immune system disorders (Nance et al. 2018). Thus, the above findings supported our conclusions that targeted therapy of microglia would have important implications for therapeutic development and precision medicine for chronic cerebral hypoperfusion. In conclusion, designing and synthesizing relevant materials for cell-type specific targets could be expected to have practical implications on ischemic injury intervention strategy.

Although the current study provides a novel dataset of hippocampus-specific gene expression profiles after 3-week cerebral hypoperfusion in mice, it also has some unavoidable limitations and deficiencies. First, our verifications were still based on the gene level, not the protein level, and the evaluation could not fully explain how these genes might ultimately affect the clinical manifestations of VCI. Future work should consist of the protein expression of the significantly different genes mentioned in the text to provide stronger evidence. Second, our transcriptomic data lacked functional level validation in the two groups of mice, which is something that can be more thoroughly considered when designing future studies. Finally, our results were somewhat descriptive. Clearly, there is still much work to be done before this basic research can be translated into clinical results.

## Conclusions

In summary, this preliminary study provides valid data for hippocampus-specific gene expression profiles following 3-week severe cerebral hypoperfusion. Our findings demonstrate that elevated levels of gene expression associated with IFN-mediated neuroimmune signaling pathways may play a crucial role in the occurrence and development of chronic ischemic injury. Our RNA-seq database may be helpful as an initial framework for future investigations of the cellular therapies in hypoperfusion-induced neuropathological changes.

## Supplementary Information

Below is the link to the electronic supplementary material.

Supplementary file1 (PDF 685 KB)

Supplementary file2 (XLSX 12 KB)

Supplementary file3 (XLSX 11 KB)

Supplementary file4 (XLSX 96 KB)

## Data Availability

The datasets provided in this study can be found in the online repository. The names of the repository/repositories and accession number(s) can be found in https://www.ncbi.nlm.nih.gov/geo/query/acc.cgi?acc=GSE223580.
