# Supplementary material for: Transcriptome Profiling of Hippocampus After Cerebral Hypoperfusion in Mice
Source: J Mol Neurosci. 2023 Jun 2;73(6):423–36. doi: 10.1007/s12031-023-02123-0 (PMC10432347; doi:10.1007/s12031-023-02123-0)

Transcriptome profiling of hippocampus after cerebral hypoperfusion in mice

Journal of molecular neuroscience

Supplemental Material

Zengyu Zhang[1,2#], Zimin Guo[1,2#], Pengpeng Jin[3], Hualan Yang[1], Mengting Hu[1], Yuan Zhang[4], Zhilan Tu[1*], Shuangxing Hou[1*]

[1]Department of Neurology, Shanghai Pudong Hospital, Fudan University, Shanghai 201399, China
[2]Shanghai Medical College, Fudan University, Shanghai 200032, China
[3]Department of Chronic Disease Management, Shanghai Pudong Hospital, Fudan University, Shanghai 201399, China
[4]Department of Vascular Surgery, Shanghai Pudong Hospital, Fudan University, Shanghai 201399, China

#These authors contributed equally to this work

***Corresponding authors**
Shuangxing Hou

housx021@163.com

Zhilan Tu

564511281@qq.com

Table S2. List of primer sequences used for qRT-PCR analysis.

**Gene**	**Forward Primer**	**Reserve Primer**
Gapdh	AATGTGTCCGTCGTGGATCTGA	GATGCCTGCTTCACCACCTTCT
Ifi211	CCAGTCACCAATACTCCACAGC	CTCTGAGTGGAGAACAGCACCT
Gm4951	GGACAAGCTGATAAGTGACCTCC	CAGCCAAGTCTTCCACCTCAGA
Ifi206	GCGAAGATATTCCAGAACTTGC	CTGGAGGTAAGTGGAGGAGG
Ifi209	CTCCAGTGTCTTCAGGAACAGC	CCTTGCTGGTGACCATTTTCCTC
Irgm1	CCTTCCCCTTTGTCACGTCATAC	CTCCTTGATTCCGTAGACCAGTT
Irgm2	ATCTCTCCACCTGGTTGCTCTG	TTCCAGTGCGATACAACCGAGC
Gbp2	ACAAGCTAGCTGGGAAGAGG	TTCAAGGCCCTCAGTGTCAA
Ifnar2	GAGCCTAGAGACTATCACACCG	TACCAGAGGGTGTAGTTAGCGG
Irf1	AACCAAATCCCAGGGCTGAT	GGAACAGACAGGCATCCTTG
Ifi204	GCCAGCCCTAAGATCTGTGA	TTTTCCACTCCCCACCACTT
Sting1	ATGTCACAGGATGCCAAAGC	ACAGTGAGAAACTGTTTCCGTC

Fig. S1 Representative images for quality control using the software FastQC. (A) Basic statistics. (B) Per base sequence quality. (C) Per tile sequence quality. (D) Per sequence quality scores. (E) Per base N content. (F) Adapter content.

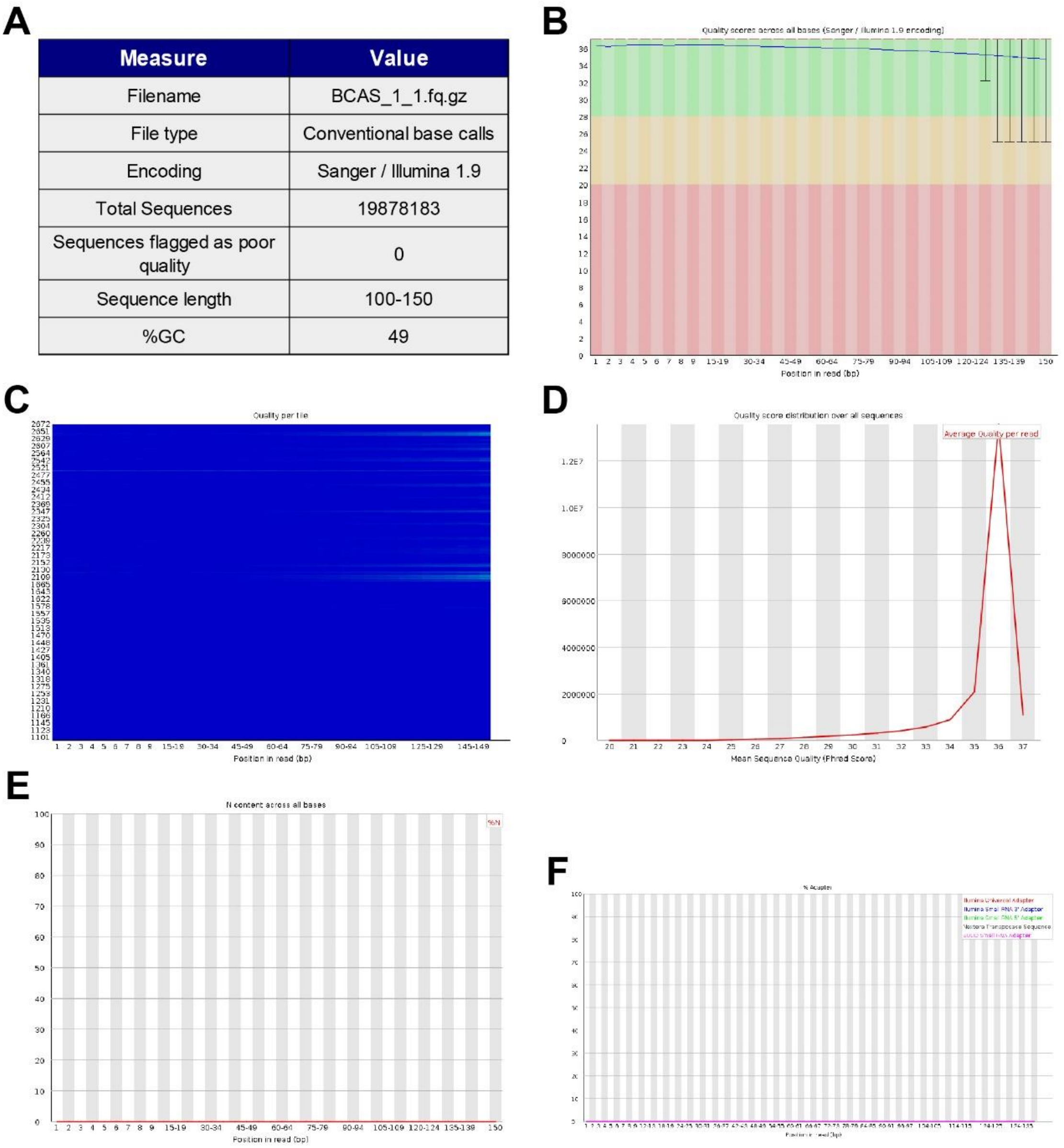

Fig. S2 Hippocampus-specific pathway analysis of transcriptome changes after BCAS-hypoperfusion. (A-D) Enrichment plots in the top four important down-regulated pathways. These include "Protein localization to synapse", "Mitochondrial respiratory chain complex I assembly", "NADH dehydrogenase complex assembly", and "Regulation of postsynaptic neurotransmitter receptor activity". Notice decreases of RNA-seq signal for genes of these pathways in BCAS group compared to sham group. NES, normalized enrichment score.

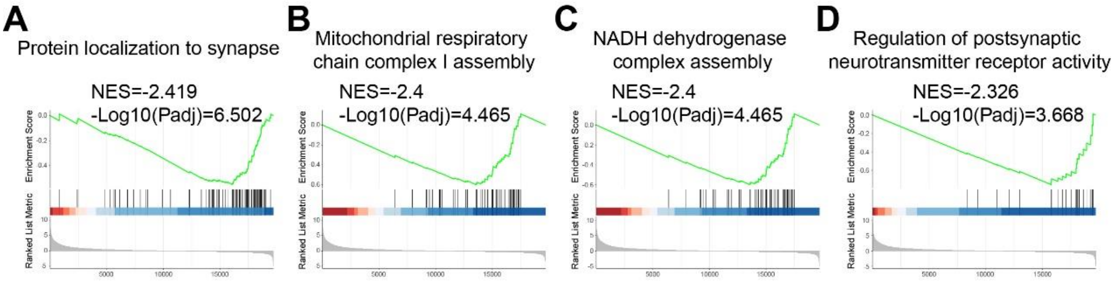

Fig. S3 Single-cell transcriptome analysis of the cell types in the mouse cortex and hippocampus. (A) The expression and percent of cell classes in the UMAP map of the published scRNA-seq dataset on the mouse somatosensory cortex (S1) and hippocampal CA1 region (GSE60361). (B) UMAP plots showing the expression level of marker genes for each cell-type of the brain (GSE60361).

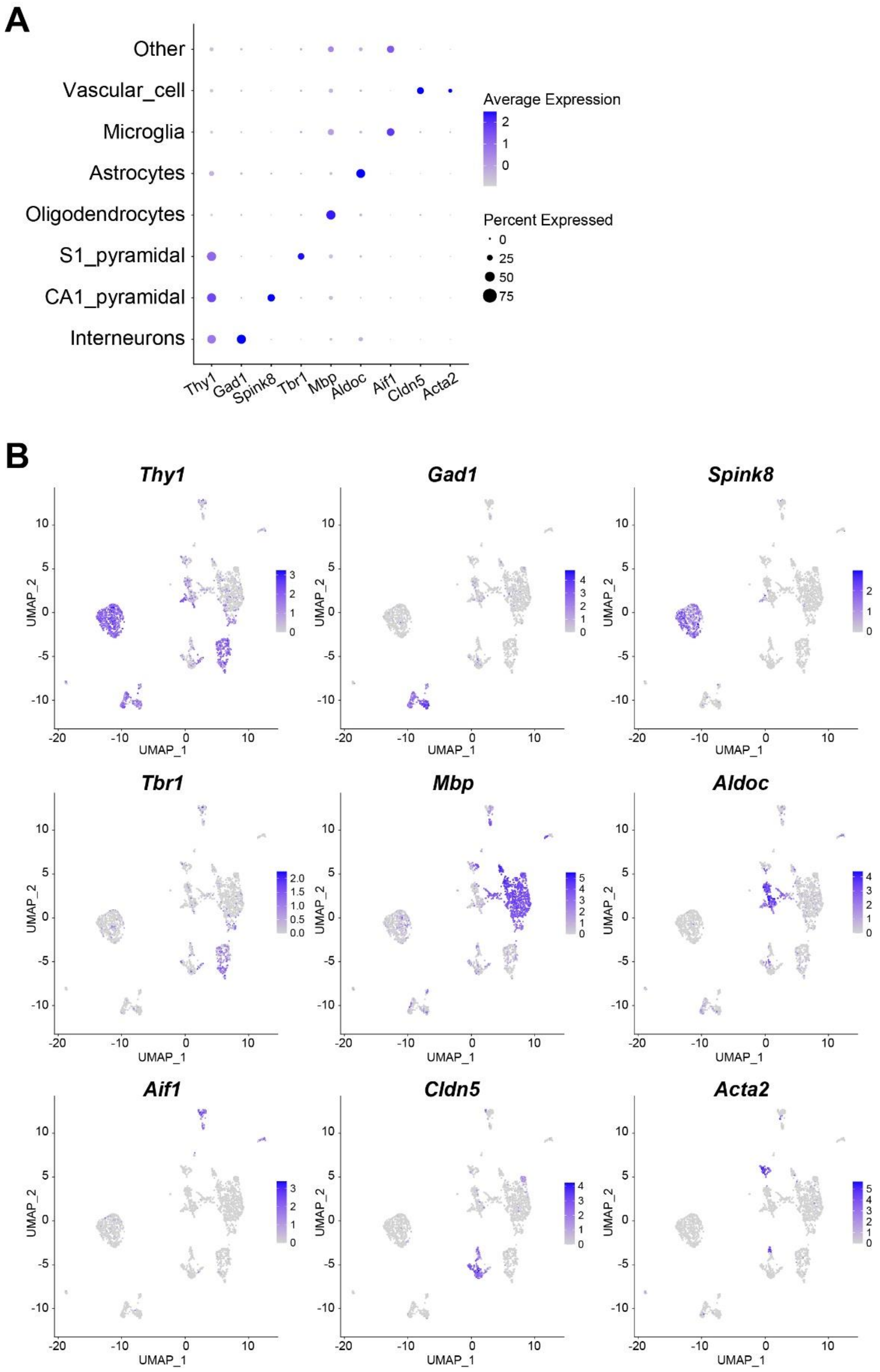

Supplement: Supplementary file 1 — Supplementary file1 (PDF 685 KB) [file 12031_2023_2123_MOESM1_ESM.pdf]
